# Mixed-Mode Crack Growth Behavior of Compact Tension Shear (CTS) Specimens: A Study on the Impact of the Fatigue Stress Ratio, Loading Angle, and Geometry Thickness

**DOI:** 10.3390/ma18071484

**Published:** 2025-03-26

**Authors:** Yahya Ali Fageehi, Abdulnaser M. Alshoaibi

**Affiliations:** Department of Mechanical Engineering, College of Engineering and Computer Sciences, Jazan University, Jazan 45142, Saudi Arabia; yfageehi@jazanu.edu.sa

**Keywords:** CTS, loading angle, fatigue life cycles, stress ratio, finite element analysis

## Abstract

The majority of engineering structures are subjected to intricate loading scenarios or possess intricate geometries, resulting in a mixed-mode stress within the component. This study aims to investigate the fracture behavior of these components under mixed-mode loading conditions by examining the relationship among the fatigue stress ratio (R), loading angle, and geometry thicknesses in compact tension shear (CTS) specimens. Using advanced ANSYS simulation techniques, this research explores how these factors affect the fatigue life cycles of engineering materials. To simulate real-world loading scenarios and study various mixed-mode configurations, compact tension shear (CTS) specimens were subjected to three specific loading angles: 30°, 45°, and 60°. These angles were applied in combination with various stress ratios (0.1–0.5) to capture a wide range of loading conditions. This study employed ANSYS Workbench 19.2, featuring cutting-edge technologies such as separating, morphing, and adaptive remeshing (SMART), to precisely model crack growth, calculate fatigue life, and analyze stress distribution. A comparative analysis with experimental data revealed that the loading angle has a profound effect on both the trajectory of fatigue crack growth (FCG) and the number of fatigue life cycles. The results demonstrate that the loading angle significantly influences the trajectory of FCG and the number of fatigue life cycles. Specifically, a loading angle of 45 degrees resulted in the maximum principal and shear stresses, indicating a state of pure shear loading. The findings reveal critical insights into the interaction between stress ratios, geometry thicknesses, fatigue life cycles, and loading angles, enhancing the understanding of engineering components’ behavior under mixed-mode stress situations.

## 1. Introduction

Fatigue failure remains one of the most significant concerns in engineering design and materials science, especially in situations where components experience repeated cycles of loading and unloading. It is a complex phenomenon influenced by various factors, including material properties, environmental conditions, and loading parameters. The significance of fatigue failure extends across multiple sectors, including aerospace, automotive, civil engineering, and biomechanics, due to its potential consequences for the structural integrity and safety of components. Structures in engineering that have defects, especially those with inclined cracks or experiencing both mode I and mode II cyclic loading, are at an increased risk of fatigue failures in mixed fracture modes. It has been reported that mode II fatigue cracks can initiate at stress levels lower than those needed for pure mode I FCG [1]. Furthermore, the rate of FCG in mixed mode I and II fatigue may sometimes exceed that of pure mode I. Thus, it is essential to conduct a thorough investigation into how mixed fracture modes affect the FCG behavior of materials [2]. The fatigue life is heavily influenced by the stress ratio, which is defined as the ratio of the minimum to the maximum stress during a single loading cycle. Understanding the factors influencing fatigue life, such as the fatigue stress ratio and loading angle, is essential for developing reliable and durable structures. Li et al. [3] found that internal crack initiation and early growth typically display a faceted appearance when the stress ratio (*R*) is greater than 0. In contrast, when *R* is equal to −1, internal crack initiation and early growth show a rough surface characteristic. Gao et al. [4] investigated the prediction of fatigue strength at varying stress ratios, focusing on damage in metallic materials, including 35CrMo steel. Their research revealed that an increase in the stress ratio results in a change in the damage mechanism, which, in turn, leads to a growth in prediction errors associated with conventional models for fatigue strength. This discrepancy occurs because these models cannot accurately capture the evolution of fatigue damage in metallic materials under certain specific conditions. In summary, Gao et al. highlighted the need for improved models that can accurately predict fatigue strength under different stress ratio conditions, taking into account the unique damage mechanisms of various metallic materials. The fatigue stress ratio (*R*) is an important factor to consider in the analysis of fatigue behavior. It influences the mean stress and the alternating stress experienced by a material during cyclic loading [5,6]. The stress ratio is a critical parameter in fatigue analysis. It influences the mean stress and the alternating stress experienced by a material during cyclic loading. A positive *R* value indicates that the material experiences tensile loading throughout the cycle, while a negative *R* value signifies that the material undergoes compressive loading [7]. Additionally, an *R* value of zero indicates a loading condition where the minimum stress during a cyclic load cycle is zero, implying that the stress fluctuates between a maximum value (tensile stress) and zero (no stress). In contrast, an *R* value of -1 signifies a fully reversed cycle, in which the material experiences equal levels of tensile and compressive loading. Cyclic loadings typically exhibit a sinusoidal pattern, characterized by minimum stress (*σ*_min_) and maximum stress (*σ*_max_) values. As an alternative representation, the cyclic loading can also be defined using the mean stress (*σ_m_*) and stress amplitude (*σ_a_*) based on the following formulas [8]:

(1) $$\sigma_{m}=\frac{\sigma_{\max}+\sigma_{\min}}{2}$$

(2) $$\sigma_{a}=\left|\frac{\sigma_{\max}-\sigma_{\min}}{2}\right|$$

where

(3) $$R=\frac{\sigma_{\min}}{\sigma_{\max}}$$

**Figure 1:**
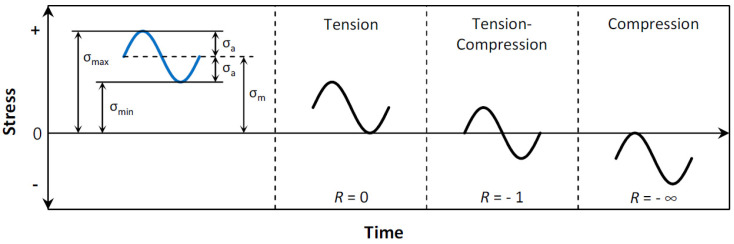
Fatigue stress cycles for different stress ratios: *R* = 0, −1, and −∞.

Figure 1 depicts these definitions alongside three illustrative stress ratios: *R* = 0, *R* = −1, and *R* = −∞.

Even with substantial knowledge gained, the role of the stress ratio on the fatigue crack growth (FCG) rate of metallic materials remains unclear [9]. Malipatil et al. [10] concluded their study by noting that for Inconel 720, a nickel-based superalloy, the FCG rate curves show a consistent trend: as the stress ratio increases, the growth rates also increase while the threshold stress intensity factor (SIF) range decreases. Numerous studies have attempted to quantify the relationship between the stress ratio and FCG rate across diverse materials [11,12,13,14]. An investigation was previously performed to understand the effects of the maximum stress intensity and load ratio (R = 0.1–0.95) on fatigue-crack propagation, specifically examining the threshold behavior in a Ti–6Al–4V alloy [15]. Bian et al. [13] investigated the fatigue crack growth of 980 high-strength steel at a stress ratio of 0.1, examining various thicknesses to assess their impact on FCG rates. They reported a notable thickness effect on the FCG behavior of the material, indicating that the fatigue crack growth life of straight-through crack specimens significantly decreases as the thickness increases. The loading angle, defined as the orientation of the applied load relative to the material’s grain structure, plays a crucial role in fatigue performance. Different loading angles can lead to varying stress distributions and crack propagation patterns within the material, influencing fatigue life significantly. For instance, loading angles aligned with the grain direction can enhance fatigue resistance, while misaligned angles may accelerate crack initiation. The CTS test configuration, introduced by researchers Richard and Benitz [16], has emerged as a pivotal tool for evaluating the FCG characteristics of materials when subjected to mixed-mode loading conditions [2,17,18,19,20]. This specialized specimen design has gained widespread adoption across various industries, as it enables a comprehensive assessment of crack behavior under the combined effects of tensile and shear stresses, which is a critical aspect in ensuring the long-term reliability and resilience of engineered structures and components. While the CTS specimen is widely used to assess FCG under mixed-mode loading (types I and II), the combined effect of the stress ratio and specimen thickness on overall fatigue life remains under-investigated. This is a critical oversight, as both parameters significantly influence the fatigue performance and durability of engineering structures and components operating under complex loading conditions. In the field of FCG analysis, a variety of software solutions are available, including ZENCRACK [21,22], ANSYS [23,24,25,26,27,28,29], ABAQUS [30,31,32,33], COMSOL [34,35], FRANC3D [36,37,38], and BEASY [39]. These tools leverage numerical simulations through the finite element method (FEM) to effectively reduce the time and costs associated with experimental testing. One notable advancement is the SMART feature in ANSYS Mechanical 19.2, which enhances the efficiency and accuracy of FCG simulations. The new feature in ANSYS Mechanical automates the processes of crack initiation and propagation, using advanced algorithms to simulate crack growth paths. This study presents a novel approach to exploring the fracture behavior of engineering components under mixed-mode loading conditions by analyzing the interaction between the fatigue stress ratio (R), loading angle, and geometry thickness in CTS specimens. Leveraging advanced ANSYS Workbench 19.2 simulations, incorporating state-of-the-art technologies such as separating, morphing, and adaptive remeshing (SMART), this research accurately models crack growth, fatigue life, and stress distribution under realistic loading scenarios. The findings reveal that optimizing these parameters significantly enhances the durability and fatigue life of structural components, with stress ratios (R) from 0.1 to 0.5 showing a notable increase in fatigue life cycles. These results offer practical guidance for engineers to improve fatigue resistance in real-world applications. While previous research has explored mixed-mode loading and fatigue behavior, a significant gap remains in understanding the combined effects of the stress ratio, loading angle, and geometry thickness on fatigue life and crack growth. This study addresses that gap through a detailed analysis supported by both simulation and experimental validation.

## 2. Numerical Analysis Procedure

ANSYS offers three crack modeling approaches: arbitrary, semi-elliptical, and pre-meshed, with the latter used by the SMART crack growth tool. This tool calculates the SIF via the crack front, a key failure criterion. SMART technology comprises three key features: first, the ability to separate and redefine complex geometric entities; second, the morphing capability, which allows for smooth and continuous deformation of these entities; and third, adaptive remeshing, which optimizes the mesh for accurate and efficient simulations during large deformations. Utilizing the Unstructured Mesh Method (UMM) with automated tetrahedral meshing (instead of hex meshes) for the crack front significantly reduces the pre-processing time and improves accuracy by enabling automatic crack front updates during simulation. This tetrahedral meshing is also employed within the SMART analysis for automatic crack front updates during crack growth. This study employed ANSYS to simulate mixed-mode loading conditions using the maximum circumferential stress as the crack growth criterion. This criterion is based on the premise that crack propagation occurs in the direction of maximum circumferential (tangential) stress at the crack surface. The ANSYS crack growth path, based on the maximum circumferential stress, is calculated using the following formula [40,41]:

(4) $$\theta =\cos^{-1}\;\left(\frac{3K_{II}^{2}+K_{I}\sqrt{K_{I}^{2}+8K_{II}^{2}}}{K_{I}^{2}+9K_{II}^{2}}\right)$$

where:

*K_I_* and *K_II_* represent the modes of SIFs, whereas *θ* represents the angle of the crack growth.

This study employs ANSYS simulation to analyze crack growth in region II, a regime characterized by a direct proportionality between the crack growth rate and maximum stress intensity factors (SIFs). The crack growth rate in this region is modeled using a modified Paris law as follows [42,43]:

(5) $$\frac{da}{dN}=C{\left(\Delta K_{eq}\right)}^{m}$$

where:

$\Delta K_{eq}$ is the equivalent SIF, and *C* and *m* are the Paris law coefficient and exponent, respectively. The equation representing the equivalent range for the SIF can be expressed as follows [44]:

(6) ΔKeq=12cos(θ2)ΔKI(1+cosθ)−3ΔKIIsinθ

## 3. Numerical Results and Discussions

**Figure 2:**
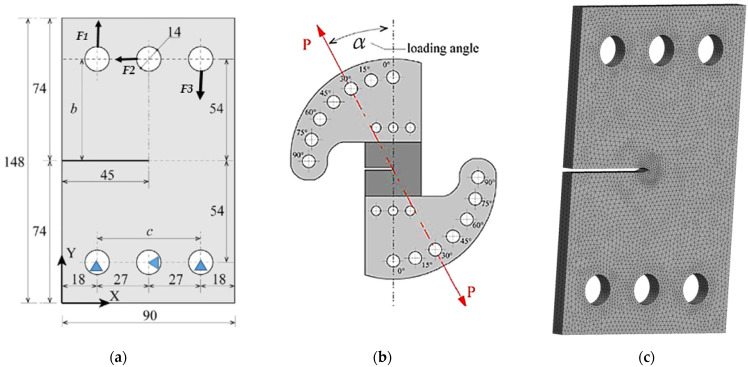
CTS: (**a**) geometrical representation and boundary condition, (**b**) loading device, (**c**) generated mesh.

A rectangular plate of AISI 316 austenitic stainless steel, measuring 90 mm in width, 148 mm in height, and 15 mm in thickness, constitutes the CTS specimen, as displayed in Figure 2a, and it aligns with the dimensions specified in reference [17]. This plate incorporates a pre-existing crack of 45 mm in length, positioned centrally along the height. At each extremity, six circular holes with a radius of 7 mm are arranged, maintaining a 27 mm distance between opposing holes. Young’s modulus (E) of AISI 316 austenitic stainless steel is 192 GPa, and its Poisson’s ratio (ν) is 0.27. The experimental results obtained by Sajith et al. [17] indicate a Paris law coefficient (C) of 4.051 × 10^−8^ and a Paris law exponent (m) of 2.348. The loading device illustrated in Figure 2b, originally developed by Richard and Benits [16], was designed to examine the crack growth behavior of CTS specimens subjected to general loading conditions. This loading mechanism comprises dual clamps that grip the CTS specimen via three bolts on both ends. Consequently, the effectiveness of this loading apparatus hinges on the specimen’s ability to undergo varied loading methods through the application of a simple tensile examination. The boundary conditions shown in Figure 2a replicate those of the loading device described by Richard and Benitz [16]. To achieve this, a rigid connector is included in the finite element model for each circular hole, ensuring no relative displacement among the edge points of the holes, making each hole behave as a rigid body. The central location of each hole, which also acts as the rotation center of the rigid connector, allows for precise application of the boundary conditions. Two sets of boundary conditions are applied: the first set, at the upper holes, consists of three external forces (*F*_1_, *F*_2_, and *F*_3_), as defined by the following formulas [45,46,47]:

(7) $$F_{1}=F(0.5\cos\alpha +\frac{c}{b}\sin\alpha )$$

(8) $$F_{2}=F\sin\alpha$$

(9) $$F_{3}=F(0.5\cos\alpha -(c/b)\sin\alpha )$$

where α denotes the loading angle (i.e., the angle of the resultant force P relative to the crack plane), while c and b, as defined in Figure 2a, are both 54 mm. The second set of boundary conditions uses kinematic constraints to fix the lower holes in place, simulating the effect of structural pins at their centers. The ANSYS-generated mesh (Figure 2c) for the CTS geometry with a 6 mm thickness comprises 156,826 nodes and 103,302 SOLID187 tetrahedral elements.

According to the foregoing Equations (7)–(9), the forces were calculated for the three loading angles, and these findings are shown in Table 1.

In the ANSYS simulation, three varying thicknesses (3 mm, 6 mm, and 15 mm) were examined for three loading angles (30°, 45°, and 60°), along with a range of stress ratios (0.1, 0.2, 0.3, 0.4, and 0.5), while maintaining a constant maximum load of 14 kN. As the loading angle increased, its influence on the crack growth trajectory became more pronounced, often leading to shear-dominated fracture modes.

**Figure 3:**
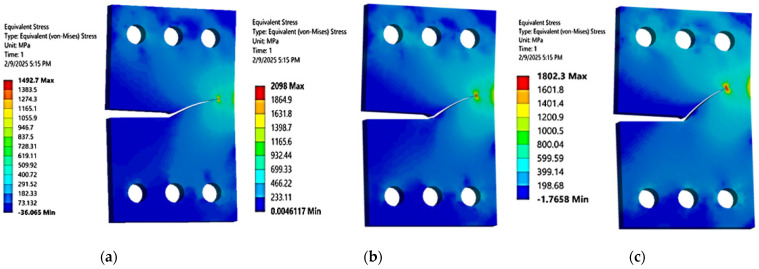
Simulated crack trajectories at different loading angles: (**a**) 30°, (**b**) 45°, and (**c**) 60°.

**Figure 4:**
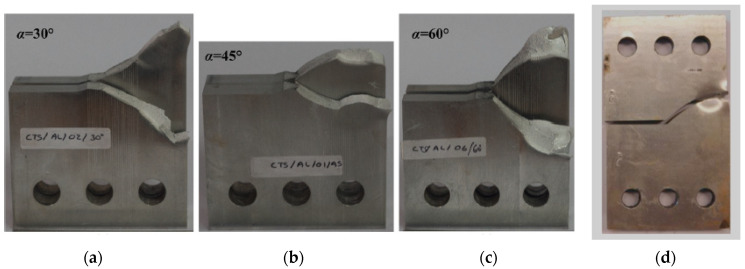
Experimental crack trajectories at different loading angles: (**a**) 30° [48], (**b**) 45° [48], (**c**) 60° [48], and (**d**) 45° [49].

**Figure 5:**
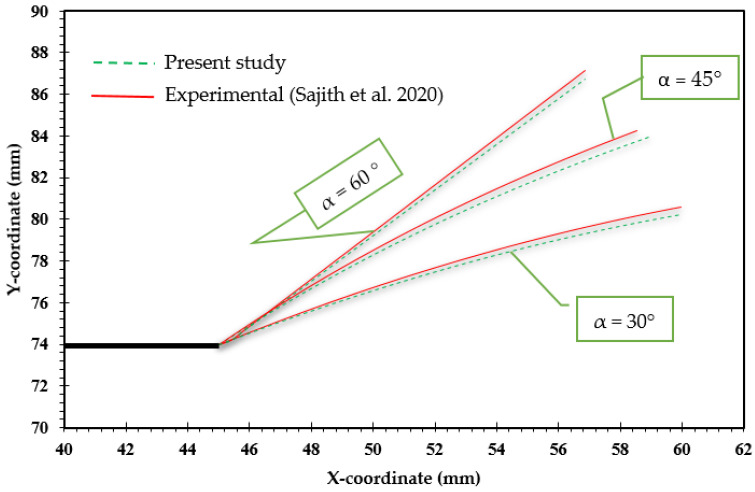
Comparison of present study’s results with experimental data from [48] across various loading angles.

In this context, Figure 3 and Figure 4 illustrate a comparison between the crack growth directions predicted from this analysis, represented by the von Mises stress distribution, and those predicted experimentally by Sajith et al. [48] across three loading angles, 30°, 45°, and 60°, and the experimental crack growth path for the loading angle of 45° predicted by [49]. To provide further quantitative assessment of the agreement between the predicted and experimental trajectories, Figure 5 depicts the crack growth paths in an X-Y coordinate system. The analysis of this figure revealed an average error of less than 0.4%.

**Figure 6:**
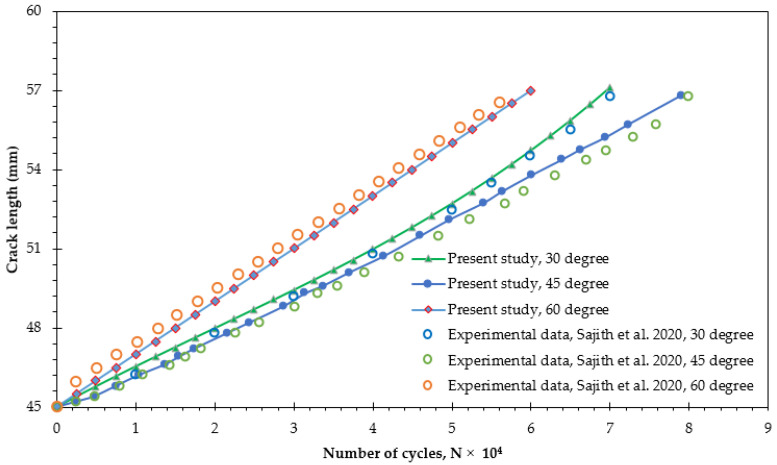
Fatigue life cycles comparison: experimental results [17,48] vs. present study across various loading angles.

The investigation involved comparing the estimated number of fatigue life cycles from the simulation for specific loading angles (30°, 45°, and 60°) with experimental results from other studies [17,48] under mixed-mode loading conditions (I/II) to ensure the accuracy of the simulation results. The significant alignment between the simulation and experimental results, depicted in Figure 6, strongly validates the precision of the fatigue life analysis in this study. This consistency also enhances the reliability of predictions for fatigue life cycles under different stress ratios, thereby deepening the understanding of the structure’s durability and its dependence on these factors.

**Figure 7:**
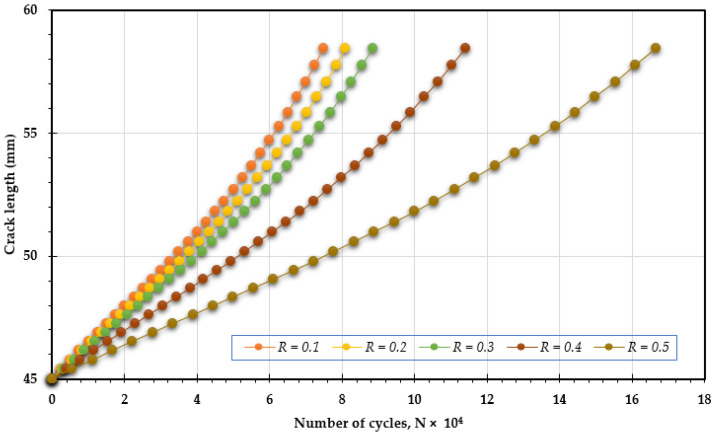
Predicted fatigue life cycles for 30° loading angle with different stress ratios.

**Figure 8:**
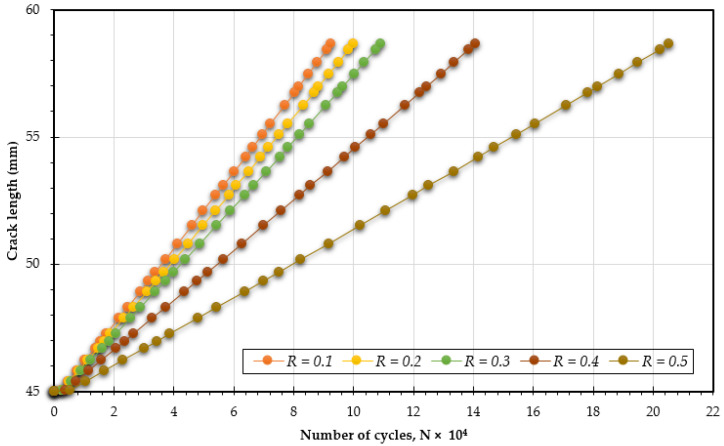
Predicted fatigue life cycles for 45° loading angle with different stress ratios.

**Figure 9:**
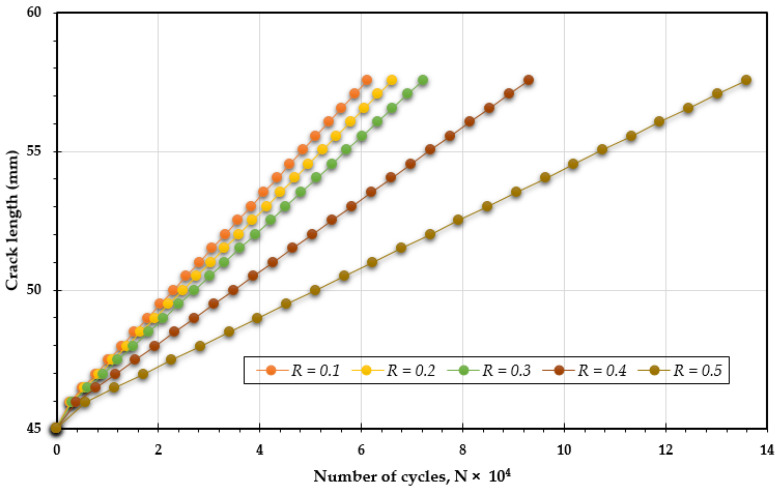
Predicted fatigue life cycles for 60° loading angle with different stress ratios.

In this analysis, Figure 7, Figure 8 and Figure 9 illustrate how changing the stress ratio (*R* = 0.1 to 0.5) influences the fatigue life across different loading angles of 30°, 45°, and 60°. The results show a significant increase in fatigue life cycles, from 7.5 × 10^4^ to 16.65 × 10^4^ for 30°, from 9.25 × 10^4^ to 21.5 × 10^4^ for 45°, and from 6.12 × 10^4^ to 13.6 × 10^4^ for 60°. The data clearly show that increasing the stress ratio is associated with a longer fatigue life. This enhancement in fatigue life can be attributed to several factors:

- Reduced Maximum Stress: A higher stress ratio results in a lower maximum stress during each loading cycle, which alleviates the overall stress experienced by the specimen. This reduction contributes significantly to prolonging the material’s fatigue life.
- Increased Compressive Cycles: A higher stress ratio leads to a larger proportion of compressive loads in each cycle, which can close existing cracks and suppress their growth, thereby increasing fatigue resistance.
- Lower Strain Rates: An increased stress ratio often leads to decreased strain rates, which can mitigate crack propagation rates, thereby extending the fatigue life.
- Minimized Plastic Deformation: Higher stress ratios tend to limit the extent of plastic deformation during loading cycles. Since plastic deformation can trigger and accelerate crack growth, reducing it plays a crucial role in enhancing fatigue life.

**Figure 10:**
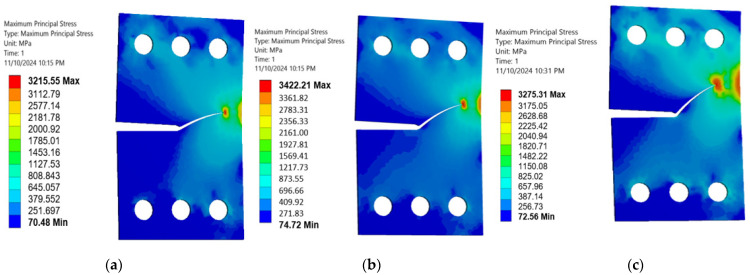
Maximum principal stress distributions in 3 mm thick CTS specimens at different loading angles: (**a**) 30°, (**b**) 45°, and (**c**) 60°.

**Figure 11:**
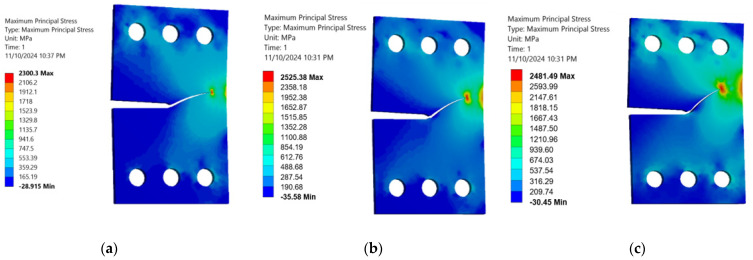
Maximum principal stress distributions in 6 mm thick CTS specimens at different loading angles: (**a**) 30°, (**b**) 45°, and (**c**) 60°.

**Figure 12:**
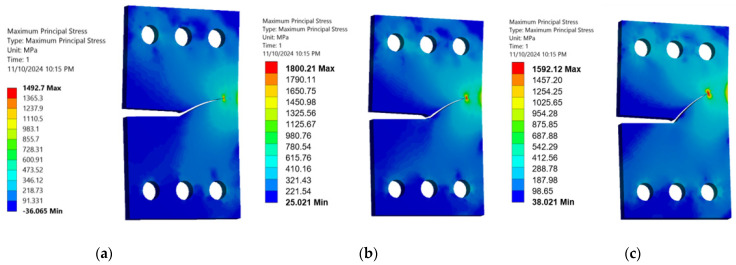
Maximum principal stress distributions in 15 mm thick CTS specimens at different loading angles: (**a**) 30°, (**b**) 45°, and (**c**) 60°.

This study analyzed how the specimen thickness influences fatigue crack growth (FCG) parameters, specifically stress and deformation, in a CTS specimen using three distinct thickness levels (3 mm, 6 mm, and 15 mm). The normal stress component peaks when the load is perpendicular to the crack plane (i.e., 0° or 90° loading angles), while the shear stress component reaches its maximum when the load is parallel to the crack plane (i.e., 45° loading angle). Figure 10, Figure 11 and Figure 12 illustrate a consistent reduction in the maximum principal stress with increasing thickness (3 mm, 6 mm, and 15 mm). These results clarify the significant effect of thickness changes on the stress–strain behavior in CTS specimens, even as the crack propagation trajectories remain unaffected. Variations in thickness cause a change in the stress–strain field, transitioning from a plane-stress to a plane-strain state as the thickness increases. This shift is associated with reduced critical fracture toughness and a smaller plastic zone. As depicted in Figure 10, Figure 11 and Figure 12, the maximum principal stress in a CTS specimen is highest at a 45° loading angle. This occurs because this angle amplifies the combined effects of both normal and shear stresses acting on the material. At loading angles of 30° and 60°, the combined stress is lower, leading to a reduced maximum principal stress. Understanding this relationship can contribute to improved material design, analysis, and selection for specific applications, ultimately enhancing the performance and safety of engineered components and systems. Moreover, the maximum principal stress can be used to predict the onset of plastic deformation and the initiation of cracks in materials, as it indicates the direction of the most significant stress concentration.

**Figure 13:**
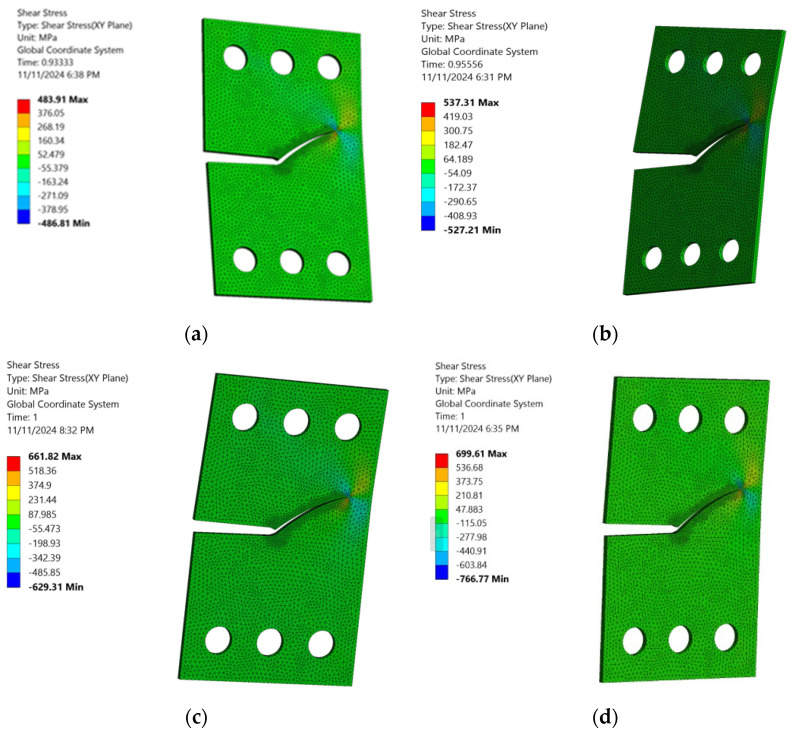
Variation in maximum shear stress distribution across different stress ratios with a loading angle of 60°: (**a**) *R* = 0.2, (**b**) *R* = 0.3, (**c**) *R* = 0.4, and (**d**) *R* = 0.5.

**Figure 14:**
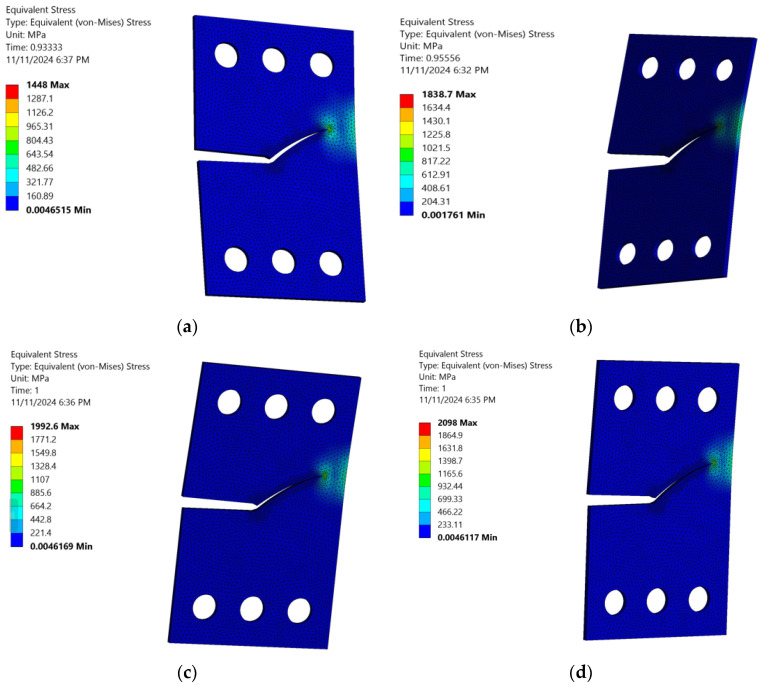
Variation in equivalent von Mises stress distribution across different stress ratios with a loading angle of 60°: (**a**) *R* = 0.2, (**b**) *R* = 0.3, (**c**) *R* = 0.4, and (**d**) *R* = 0.5.

Figure 13 and Figure 14 highlight a noteworthy observation: as the stress ratio increases, the material’s capacity to endure higher shear stress levels and greater von Mises stress levels also rises, thereby extending fatigue life cycles. This noteworthy behavior can be attributed to the material’s response to the rising stress ratio, which triggers a redistribution process that enhances its strength. Subsequently, the material becomes more resistant to fatigue damage, leading to a longer fatigue life. This novel phenomenon, where CTS specimens can tolerate greater shear stress and higher von Mises stress and experience prolonged fatigue life cycles at higher stress ratios offers engineers a valuable advantage in material selection and predicting performance under cyclic loads. This is particularly beneficial in scenarios where fatigue life and damage resistance are paramount factors. By leveraging this knowledge, engineers can optimize material choices and improve predictions of performance, thereby enhancing safety, reliability, and efficiency in structural design.

**Figure 15:**
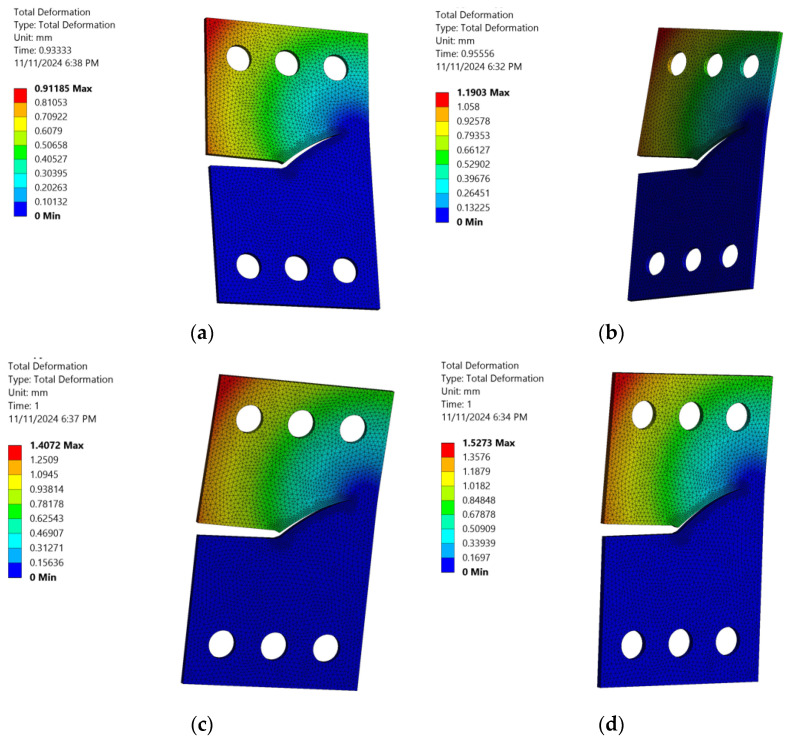
Total deformation variation across different stress ratios with a loading angle of 60°: (**a**) *R* = 0.2, (**b**) *R* = 0.3, (**c**) *R* = 0.4, and (**d**) *R* = 0.5.

When exploring the total deformation of a CTS specimen across various loading angles, it is important to consider the effect of the fatigue stress ratio on the total deformation. As this ratio increases, the CTS specimen experiences a series of factors that contribute to the increase in total deformation, as depicted in Figure 15. Specifically, this result demonstrated that increasing the fatigue stress ratio in CTS specimens leads to higher mean stress levels and reduced stress variation, resulting in greater accumulated deformation over time. Additionally, the reduced stress variation is a result of the smaller difference between the minimum and maximum stress values in each cycle, which leads to a more constant and steady stress application. This, in turn, promotes incremental and consistent deformation. This insight highlights the critical role of the fatigue stress ratio in influencing deformation behavior, crack initiation, crack growth, and fatigue life, providing a foundation for more accurate fatigue performance assessments and improved material design under cyclic loading conditions. It is important to note that the fatigue stress ratio not only affects the deformation behavior of CTS specimens but also has implications for the overall mechanical response of the material.

**Figure 16:**
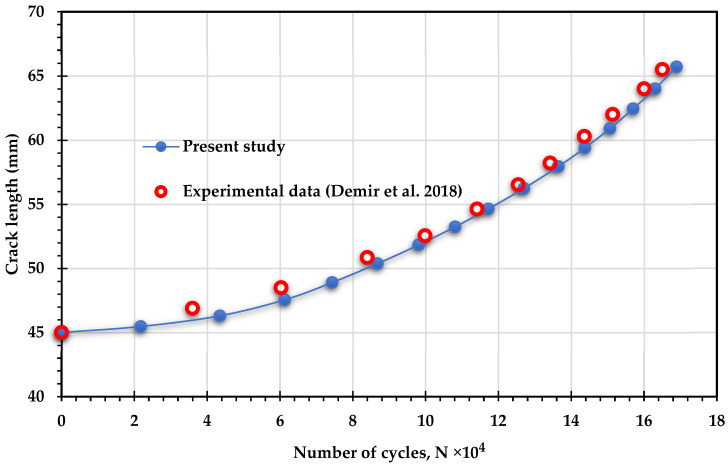
Comparison of fatigue crack growth lives predicted by ANSYS and experimental data obtained by Demir et al. [50] for 30° loading angle.

**Figure 17:**
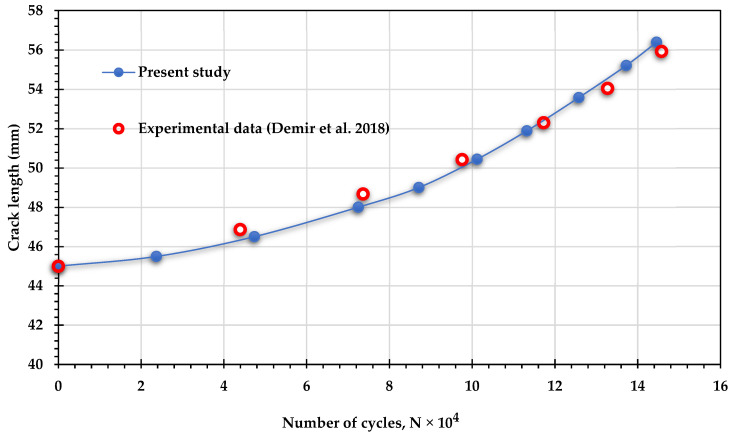
Comparison of fatigue crack growth lives predicted by ANSYS and experimental data obtained by Demir et al. [50] for 45° loading angle.

**Figure 18:**
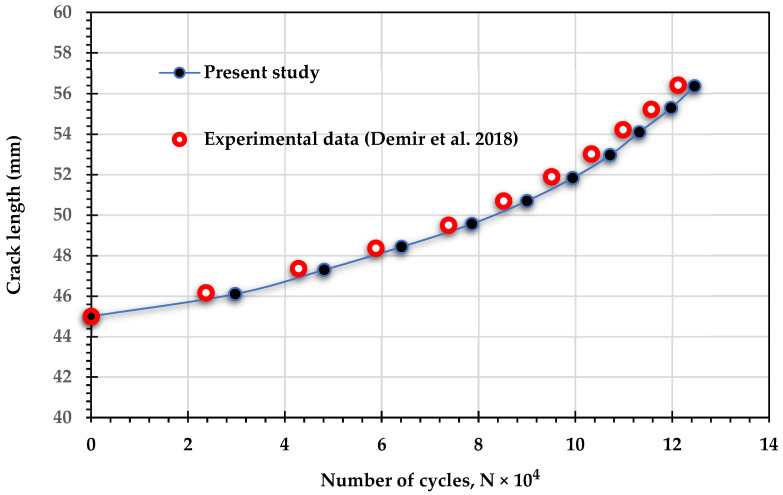
Comparison of fatigue crack growth lives predicted by ANSYS and experimental data obtained by Demir et al. [50] for 60° loading angle.

To further validate the findings of this study, the experimental results for fatigue life cycles reported by Demir et al. [50] were compared with the results obtained in the present study for three loading angles: 30°, 45°, and 60°. In their study, Demir et al. [50] used Al 7075-T651 aluminum alloy, which has an elastic modulus of 70 GPa, a Poisson’s ratio of 0.33, and a yield stress of 460 MPa. The applied loads were 11 kN for the 30° loading angle, 11.4 kN for the 45° loading angle, and 13.65 kN for the 60° loading angle, with a stress ratio of *R* = 0.1 for all cases. Figure 16, Figure 17 and Figure 18 show the comparison between the predicted fatigue life cycles in the present study and the experimental data obtained by [50], demonstrating excellent agreement. This comparison reinforces the reliability and accuracy of the current findings and provides a robust foundation for future research and practical applications in fatigue analysis.

## 4. Conclusions

The scientific contribution of this study lies in its comprehensive investigation of the complex interplay between fatigue stress ratios, loading angles, and geometry thickness on fatigue crack growth (FCG) behavior in engineering materials under mixed-mode loading conditions. By using ANSYS Mechanical for simulation and validating the results against experimental data, this research provides robust insights into how these factors influence the mechanical response of materials, particularly highlighting the significant role of the loading angle and stress ratio in determining FCG trajectories and fatigue life cycles. This study compared its simulation results to experimental data from other researchers at loading angles of 30°, 45°, and 60° to validate its simulation accuracy. The following conclusions can be drawn from this study:

- The findings reveal that the loading angle has a significant effect on the trajectory of FCG and the number of fatigue life cycles.
- This research elucidates the critical role of the fatigue stress ratio in the mechanical response of materials, demonstrating that an increased stress ratio markedly enhances fatigue life cycles. This finding provides new insights into how stress conditions affect material durability. The results demonstrate that increasing the fatigue stress ratio in CTS specimens results in higher mean stress levels and a decrease in stress variation. Consequently, this leads to greater accumulated deformation over time.
- By examining the intricate interactions among the fatigue stress ratio, loading angle, and geometry thickness, this research offers valuable guidance for optimizing component performance and safety under cyclic loading. These findings are essential for advancing design strategies, improving material selection, and enhancing life cycle assessments of fatigue-sensitive components, ultimately contributing to greater reliability and safety across various industries.

## Figures and Tables

**Table 1:** Force values for different loading angles.

α	*F*_2_	*F*_1_	*F*_3_
30	0.5 *F*	0.933 *F*	−0.067 *F*
45	0.707 *F*	1.061 *F*	−0.354 *F*
60	0.866 *F*	1.116 *F*	−0.616 *F*

## Data Availability

The original contributions presented in this study are included in the article. Further inquiries can be directed to the corresponding author.
